# Role of Tartaric Acid in Chemical, Mechanical and Self-Healing Behaviors of a Calcium-Aluminate Cement Blend with Fly Ash F under Steam and Alkali Carbonate Environments at 270 °C

**DOI:** 10.3390/ma10040342

**Published:** 2017-03-25

**Authors:** Tatiana Pyatina, Toshifumi Sugama

**Affiliations:** Brookhaven National Laboratory, Upton, NY 11973-5000, USA; sugama@bnl.gov

**Keywords:** calcium aluminate cement, self-healing cement, alkali activated cement, fly ash, retardation, microstructure

## Abstract

Tartaric acid (TA) changes short-term mechanical behavior and phase composition of sodium-metasilicate activated calcium-aluminate cement blend with fly ash, type F, when used as a set control additive to allow sufficient pumping time for underground well placement. The present work focuses on TA effect on self-healing properties of the blend under steam or alkali carbonate environments at 270 °C applicable to geothermal wells. Compressive strength recoveries and cracks sealing were examined to evaluate self-healing of the cement after repeated crush tests followed by two consecutive healing periods of 10 and 5 days at 270 °C. Optical and scanning electron microscopes, X-ray diffraction, Fourier Transform infrared and EDX measurements along with thermal gravimetric analyses were used to identify phases participating in the healing processes. Samples with 1% mass fraction of TA by weight of blend demonstrated improved strength recoveries and crack plugging properties, especially in alkali carbonate environment. This effect was attributed to silicon-rich (C,N)-A-S-H amorphous phase predominant in TA-modified samples, high-temperature stable zeolite phases along with the formation of tobermorite-type crystals in the presence of tartaric acid.

## 1. Introduction

Self-healing of cementitious materials has been explored in civil engineering during the last twenty years [1,2]. It is of particular interest for cases where the damaged areas are difficult to locate and reach [3]. High-temperature, aggressive environments of geothermal wells impose most difficult conditions on cementitious materials that must provide durable zonal isolation under repeated thermal, chemical and mechanical stresses [4]. Damaged cement sheath can lead to compromised zonal isolation, casing corrosion and, in worst cases, loss of the well. Economical cements that can recover their properties without human intervention are especially attractive for such applications.

Although research on self-healing cementitious materials evaluates various innovative venues, such as encapsulation and utilization of hollow fibers [5,6,7,8,9], intrinsic self-healing continues to be more economical and robust compared to other technologies [10]. Such self-healing was observed in civil structures long time ago [11]. 

Well cements have several advantages in terms of the conditions that may be favorable for intrinsic self-healing. These typically include exposure to well fluids that may noticeably promote self-healing in cementitious materials [12,13,14,15]; cement sheath is constrained by the wellbore and casing that may limit displacement of the cracked and fractured species minimizing the size of fractures, which is one of the conditions for successful self-healing [16,17,18]; and presence of other ions in the wellbore and formation fluids that may participate in the reactions with the set cement to form new products favoring cracks sealing and/or strength recoveries. In addition, high temperatures of subterranean environments, which limit applications of temperature sensitive materials such as polymers and bacteria, promote pozzolanic reactions even at low residual alkalinity of partially reacted cementitious matrix when fluids penetrate through fractures and cracks allowing cracks closure and matrix healing by the new reactants.

The principal methodologies in intrinsic cement healing include hydration, swelling and expansion of non-hydrated cementitious components and latent reactions of secondary cementitious materials such as different types of fly ash, slags, clays, geo-materials and other pozzolanic materials such as glass [3,19,20,21,22]. These healing mechanisms apply mostly to the young cements at early hydration times; however, recoveries of mechanical properties were also reported for cements cured for 180 days where most hydration reactions were stabilized [15]. Other reported healing causes that may take place at later hydration times include formation of calcium carbonate in contact with atmospheric carbon dioxide or dissolved carbonates, reported years ago [23,24,25]; re-crystallization of hydrated matrix components [26]; and mechanical plugging of broken off cement pieces or originally present in the fluids particles [27]. Some researchers found newly formed C-S-H gels along with calcium carbonate in self-healed cement composites with secondary cementitious materials such as fly ash F and slag [13,15].

Several additives enhancing self-healing capacities of cements were reported. Lightweight aggregates were shown to contribute significantly to the self-healing process assessed by rapid chloride penetration method [28]. Other cement healing-enhancing additives involved calcium sulfoaluminate based expansive agents [26]; limestone powder [21] and hydrated or quick lime [7,29]; nanoparticles of functionalized silica [30] and nanoclay [21]; and reactive MgO [7,31,32]. Repeatedly, silicon-containing compounds in the form of glass [22,33,34,35], sand, fume [36] or alkali silicates such as microcapsules [6,30] or encapsulated liquids of sodium silicate, colloidal silica, tetraethylorthosilciate [32] are used as healing-enhancing additives. They have the advantage of being naturally compatible with the cement matrix and allowing both sealing the cracks and strength recovery. Among the reported self-healing enhancing additives, so-called crystalline admixtures obtained special attention in recent years. A crystalline admixture generally includes a proprietary mix of active chemicals in cement and sand [37]. Efficacy of crystalline admixtures as promoters of self-healing in the presence of water was demonstrated by several researchers [36,38,39,40,41]. Tricalcium-silicate and calcium hydroxide [39] may react with the crystalline admixtures forming modified C-S-H gel and calcium-containing, pore-blocking precipitate [37]. 

Silica-rich gel formation along with cement carbonation was suggested to help to decrease porosity in experiments with oil-field cement in a flow-through apparatus with CO_2_ saturated brine [42].

An earlier work [43] evaluated self-healing performance of Thermal Shock Resistant Cement (TSRC), formulated with calcium aluminate cement and fly ash, type F activated with sodium metasilicate activator to withstand high temperature variations encountered in geothermal environments. The cement recovered more than 86% of compressive strength and demonstrated complete crack sealing after repeated compressive damage when healed for total of 20 days in 0.05 M solution of Na_2_CO_3_ at 270 °C. In this work, carbonation of cement matrix resulted in calcium dissolution as calcium bicarbonate and formation of cancrinite, with calcium partially or completely replaced by sodium, in the cement matrix. Cancrinite helped strength recoveries while high-temperature stable zeolites along with mostly amorphous silica and silica-aluminum-hydrate gel participated in the sealing of the cracks. In the case of TSRC, sodium-silicate activator played an important role in latent pozzolanic reactions and in formation of silica-rich gel and zeolite phases filling the cracks. In the steam environment, calcium-containing feldspar minerals anorthite and dmisteinbergite were stable whereas zeolites and silica presence in the cracks was noticeably inferior to that found in alkali carbonate; this resulted in lower average strength recoveries (84%) and poorer cracks’ filling. 

The present paper evaluates the effect of tartaric acid (TA) on TSRC’s healing efficiency under alkali carbonate and steam environments at 270 °C. TA was identified as an efficient set retarder of TSRC and was shown to promote higher porosity but smaller pore size and higher compressive strength, limited number of phase transitions and preferential formation of phases stable under the final curing conditions (after short curing periods of up to three days at temperatures of up to 300 °C) [44]. Similar to crystalline additives, addition of TA modified the gel phase and resulted in preferential formation of zeolites shown to participate in the sealing of matrix fractures, so would be desirable for enhanced filling of the cracks. TA interactions with calcium could improve fractures sealing under steam conditions. Furthermore, the use of the retarder may help to spare some latent self-healing capacity for later times. 

## 2. Materials and Methods

### 2.1. Materials

FAF was obtained from Boral Material Technologies, Inc. A sodium metasilicate granular powder, under the trade name “Metso Beads 2048 (PQ Corporation)”, was used as the alkali activator of FAF. Secar #80 (Kerneos Inc.) was used as the calcium-aluminate cement. The X-ray powder diffraction (XRD) data showed that the crystalline compounds of FAF consisted mainly of quartz (SiO_2_), mullite (3Al_2_O_3_·2SiO_2_), and hematite (Fe_2_O_3_), and CAC included three crystalline phases, i.e., corundum (α-Al_2_O_3_), calcium monoaluminate (CaO·Al_2_O_3_, CA), and calcium dialuminate (CaO·2Al_2_O_3_, CA_2_). Table 1 shows the elemental composition of the TSRC components (major oxides composition measured by EDX and normalized to 100%).

The blend cement consisted of 60% CAC and 40% FAF (mass fractions). The sodium metasilicate was dry-blended with this mixture at 6.0% mass fraction of the cement blend to form the TSRC. The water-to-solid ratio of the slurries was 0.52. 

D-(-)-tartaric acid (TA) was supplied by Sigma-Aldrich. The retarder was dry-blended with half of the TSRC samples at mass fraction of 1% of the TSRC blend. The samples modified with TA are further referred to as “TA-modified” or “TA samples”

To improve toughness of the samples for repeated compressive damage tests carbon micro fibers of 7–9 µm in diameter and 100–200 µm in length (Asbury Carbons, AGM 94 grade, Asbury, NJ, USA) were added to all the formulations at 10% mass fraction by total weight of solid blends [45].

### 2.2. Samples Preparation and Testing

To prepare samples for compressive damage/strength tests, the slurries were mixed by hand at water-to-blend ratio of 0.5 and cast into cylindrical molds (25-mm diameter and about 40-mm length). Borosilicate glass tubes (25 mm × 150 mm cylinders) were used as the molds. The walls of the tubes were covered with Teflon sheets to avoid cement slurry contact with glass. The tubes were filled with the slurry up to about 40 mm height. The final samples of cement were measured before mechanical testing and the exact size of each sample was used to calculate the compressive strength. The molds were placed into an autoclave partially filled with water (20% of volume) at 85 °C for a 3-day curing, and then the temperature was increased to the final 270 °C for 24 h. The preliminary low curing temperature imitated placement conditions in geothermal wells. The volumetric proportion of cement-to-fluid was 1-to-3.5 and the pressure in the autoclaves was 8.27 MPa. The fluid used as a curing environment was either pure water or 0.05 M solution of sodium carbonate. Mechanical properties of the samples cured for 3 days at 85 °C and 24 h at 270 °C were determined in crush tests that were stopped within no more than 40% of compressive strain after the yield point [43]. Damaged samples were returned into the autoclaves and cured for 10 more days at 270 °C. Then, the crush tests were repeated to evaluate strength recoveries and damage the samples for the second time. The second-time damaged samples were cured for 5 more days under the same conditions followed by the final crush tests. Each result was averaged over at least three samples. The experimental steps are summarized below.

3 days at 85 °C ➔ 24 h at 270 °C ➔ 1st crush test ➔ 10 days at 270 °C ➔ 2nd crush test ➔ 5 days at 270 °C ➔ 3 days crush test

The sealing of the cracks was visualized using optical microscope and the elemental composition of the sealing phases analyzed with µEDX after the first 10 days of crack healing at 270 °C. The morphologies of the cements were studied on typical spots of freshly fractured samples after total of 15 days of healing with a JEOL 7600F scanning electron microscope (JEOL, USA Inc., Peabody, MA, USA) equipped with an EDX Oxford Link microanalysis system. The specimens were placed on metallic holders and coated with chromium.

After the specimens were tested for strength and crack sealing, samples were separated into cores and materials scratched out of the crack areas. They were ground into fine powder and dried at 90 °C for 24 h prior to thermogravimetric, Attenuated Total Reflectance-Fourier Transform Infrared Spectroscopy testing (FT-IR Spectrometer Spectrum 100, Perkin Elmer) and Philips X-ray diffractometric characterization. The former analysis was done on approximately 10 mg of sample heated at a rate of 20 °C /min in N_2_, using TGA-Q50 (TA Instruments). The samples were examined using an X-ray diffractometer with a 40 kV, 40 mA copper anode X-ray tube. The results were analyzed using the PDF-4/Minerals 2015 database of the International Center for Diffraction Data.

## 3. Results

### 3.1. Mechanical Properties

One approach to obtaining information on the self-healing ability of defected cement is the measurement of the original strength recovery. The original compressive strength of TA-modified and control cement samples was measured after curing in water or alkali carbonate for 24 h at 270 °C. It was compared against that measured after the repeated crush tests and two curing periods. The focus was on evaluating whether the materials damaged at early times, when the probability of both mechanical and thermal stresses in geothermal wells is high, may somewhat recover their properties and still provide well integrity. This is an important point since the decisions on very costly and difficult interventions for well-repairs could be based on well evaluations done at early times in the well-life without taking into consideration possibility of materials healing. That is the reason why the early (24 h) strengths of the non-damaged samples were used as control values for healed specimens.

**Figure 1:**
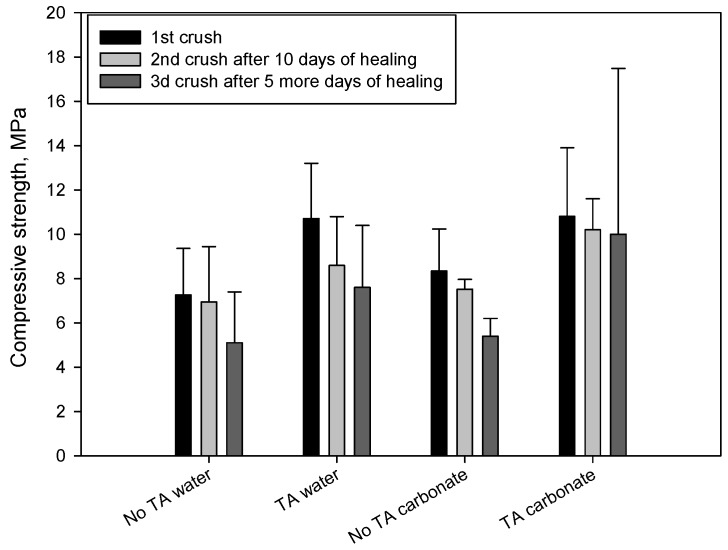
Compressive strength for the first-, second- and third-time crushed TA-modified and control samples after exposure to water or alkali carbonate solution at 270 °C.

Figure 1 gives the strength values of the first-, second- and third-time crushed samples under the compressive load. The data for the first crush (original strength) revealed that TA-modified samples displayed a better development of compressive strength at the curing age of 24 h than control cement. In fact, the compressive strength of 10.7 and 10.8 MPa, respectively, for TA samples cured in water and carbonate, was 32% and 23% higher than that of control samples. The value of compressive strength for all first-time crushed samples after the first 10 days of healing in these environments was somewhat lower, compared with that of the original strength, correspondingly, the rate of strength recovery ranged from 80% to 95%. After the healing exposure to the test environments was extended for 5 more days followed by the crush strength tests, the average strength recovery rates after 10 days and additional 5 days were evaluated. Although the data dispersion was high for the third-time crushed TA-samples cured in carbonate the general trend showing better strength recoveries of samples modified with TA is clearly seen. Thus, TA offered the improved cement strength recovery. 

### 3.2. Crack Sealing, Optical Microscope and µEDX Elemental Imaging

**Figure 2:**
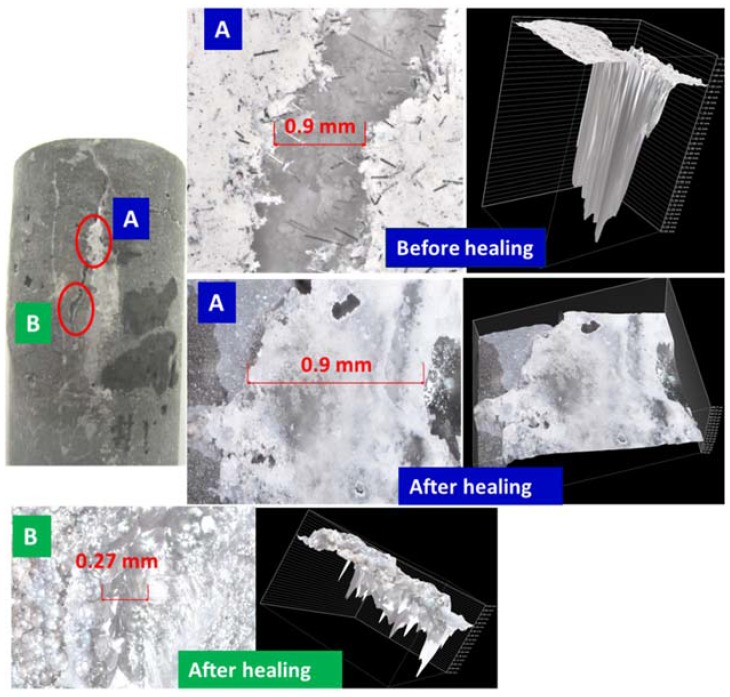
Optical microscope images of cracks in damaged control samples before and after healing in water for 10 days at 270 °C.

**Figure 3:**
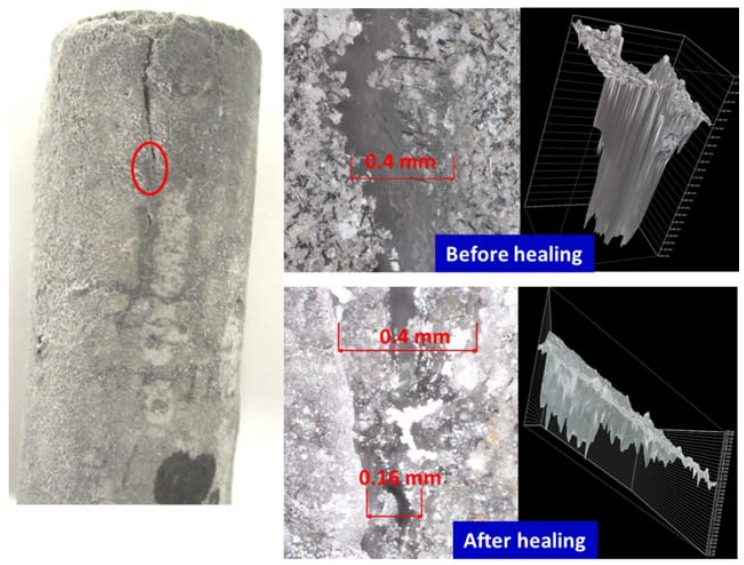
Optical microscope images of cracks in damaged control samples before and after healing in alkali carbonate for 10 days at 270 °C.

**Figure 4:**
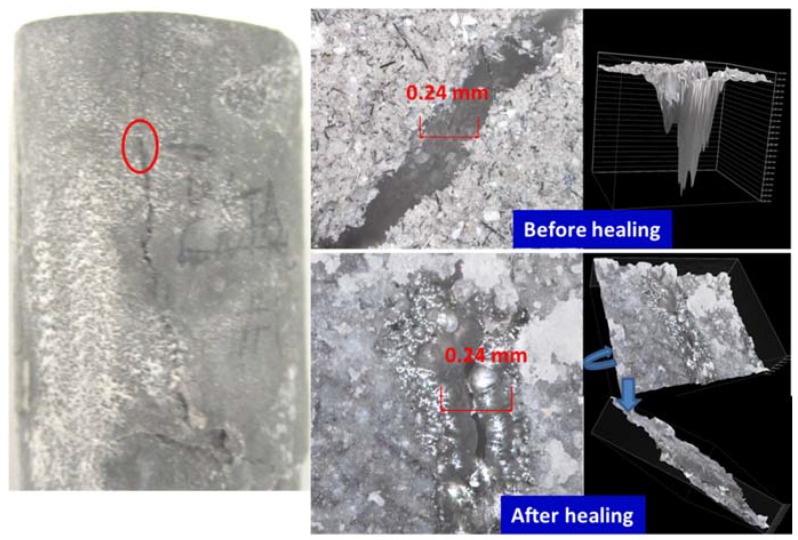
Optical microscope images of cracks in damaged TA-modified samples before and after healing in water for 10 days at 270 °C.

**Figure 5:**
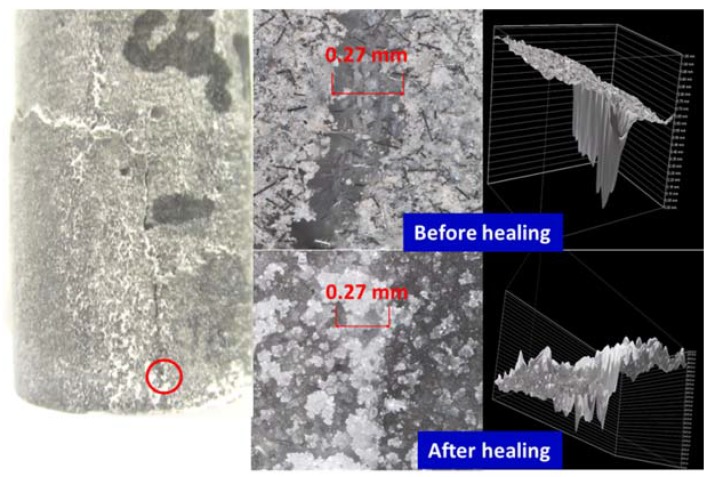
Optical microscope images of cracks in damaged TA-modified samples before and after healing in alkali carbonate for 10 days at 270 °C.

Figure 2, Figure 3, Figure 4 and Figure 5 show optical microscope images of control and TA-modified samples exposed to water or carbonate environments after the crush tests. The photographs and the corresponding 3D images (images with the black background to the right of the photographs) show the same cracks before and after the healing (the “before” image for the crack in location B of Figure 2 is not shown). 

All samples underwent some crack sealing. The nature of the healing material visually differed depending on the curing environment. The water-cured samples showed a smooth gel-like (glassy in the case of the control) filling, while it was rougher in the alkaline carbonate especially for TA-samples.

Impressively, the gel completely sealed the 0.9 mm wide and 1.75 mm deep crack and partially filled a narrower 0.27 mm crack in some crack areas of the control sample (Figure 2). The 3D images show the flat gel surface covering the 0.9 mm crack. Similarly, for the TA-modified sample, the 0.24 mm wide and 1.1 mm deep crack was totally closed with a solid gel (Figure 4). The 3D image is shown from two angles to demonstrate the absence of the depth in the healed crack area. 

The sealing was more efficient for TA-modified samples than for control in alkaline carbonate. For example, 0.16 mm crack in control sample was only partially healed, while 0.27 mm crack in TA-modified sample was sealed completely (Figure 3 and Figure 5). The sealing material was rougher with a visible build up above the crack area for this environment. Unlike the gel formed in water, this filling did not cover the cracks but filled them and the healing was lesser for the deeper/wider fractures. 

The results of the elemental composition mapping for the cores of the samples and the healed crack areas are given in Table 2. The data were obtained by grinding samples collected from cores or scratched out of the healed crack areas.

Comparison of the core and crack compositions can provide information on the elements preferentially participating in the healing process. The data presented in Table 2 clearly indicate silicon-rich material accumulation in the crack areas. Concentrations of SiO_2_ in the cracks are 3.1–3.5 times higher than in the cores of the samples. Accordingly, the CaO/SiO_2_ and Al_2_O_3_/SiO_2_ ratios in the ranges 0.78–0.91 and 2.2–2.4 in the cores drop to 0.04–0.07 and 0.14–0.27, respectively, in the healed cracks. 

The CaO/Al_2_O_3_ ratio of control samples did not change significantly in water environment and increased in carbonate, possibly as a result of calcium carbonate precipitation in the cracks. TA-modified samples seemed to have lower calcium and higher aluminum contents in the healed areas than in the cores, which resulted in lower CaO/Al_2_O_3_ ratio for the crack material. The lesser drop in Al_2_O_3_/SiO_2_ ratio in the sealed areas for TA-modified samples than for controls is in agreement with this observation. Elemental mapping of aluminum (not shown) demonstrated that it was present in low amounts throughout the crack areas in both environments.

In summary, the micro-images show that both control and TA-modified samples undergo sealing of the fractures accompanied by the increase of silicon surface content and in the case of TA-modified samples some increase in aluminum in the crack sealing material. TA enhances sealing of the cracks especially in carbonate environment. 

### 3.3. XRD Study

**Figure 6:**
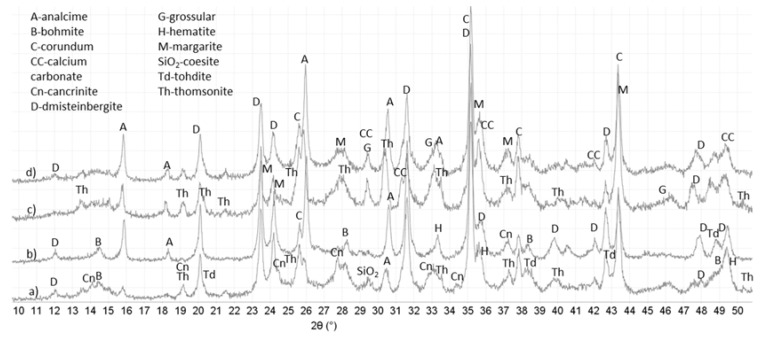
Diffractograms for control and TA-modified TSRC samples after total of 15 days in water at 270 °C: (**a**) control-core; (**b**) control-cracks; (**c**) TA-core; and (**d**) TA-cracks.

**Figure 7:**
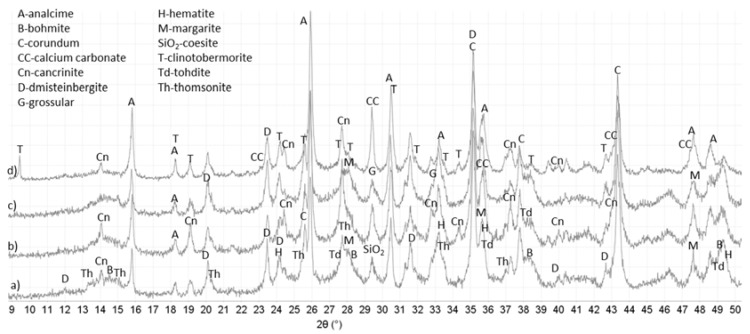
Diffractograms for control and TA-modified TSRC samples after total of 15 days in alkali carbonate at 270 °C: (**a**) control-core; (**b**) control-cracks; (**c**) TA-core; and (**d**) TA-cracks.

Figure 6 and Figure 7 show major crystalline phases for control and TA-modified samples cured in water or alkali carbonate at 270 °C for total of 15 days and Table 3 lists these phases with their respective ICDD numbers. The phases appear in the table in the order of higher to lower abundance according to the semi-quantitative analyses of the PDF-4/Minerals 2015 database. The major crystalline phases were for the most part similar in control and TA-modified samples after total 15 days of curing. In addition to the listed phases all the samples showed peaks of corundum, some hematite (from non-reacted fly ash) and the crack areas had some calcium carbonate-related minerals (calcite and valerite).

The major crystalline reaction products for all samples included: feldspar-type mineral dmisteinbergite; mica-type mineral margarite-2M1; zeolites thomsonite with varied calcium content and analcime. In the case of the control aluminum oxide hydroxides bohmite and tohdite (split bohmite peaks at 2θ 38.3° and peak shoulders at 20.3° and 42.9°) along with the small amounts of feldspathoid group mineral cancrinite were mostly present in the core of the sample. 

For the TA-modified cement, additional crystalline phases included garnet-group mineral grossular in water-cured samples and clinotobermorite (or distorted tobermorite) that gave a visible low-angle peak at 2θ 9.43° in the crack areas of TA-modified sample cured in alkali carbonate. This phase was absent from all other samples. Small peaks of paragonite, sodium-aluminum silicate, mica-type mineral appeared in cracks of the TA-sample cured in alkali carbonate. 

Although the major crystalline phases were similar in both control and TA-modified samples there were noticeable differences in their manifestation. The same mineral phases including predominantly dmisteinbergite, margarite, grossular and aluminum oxide hydroxides formed in the cores of control and modified samples. However, water-cured control samples showed higher core intensities of dmistenbergite (peaks at 2θ°: 23.5, 24.1, 31.6, 35.3) and aluminum oxide hydroxides (peaks at 2θ°: 14.52, 28.25, 38.34 and between 48.93 and 49.34) while TA-modified ones were richer in margarite (2θ°: 35.6) and zeolite thomsonite (peaks at 2θ°: 13.4, 15.0, 30.45, 33.6). 

For the crack zones, the major phases included zeolites and cancrinite for both control and modified samples. However, TA-samples crack areas were richer in zeolites (analcime and thomsonite). Thus, the intensities of analcime’s peaks (2θ° 15.8) were more important for TA-samples and thomsonite was present only in the patterns of water-cured TA-modified cement. In contrast, cancrinite (a likely carbonation product of dmisteinbergite) forming mostly in carbonate environment had higher peak intensities in control samples than in TA-modified ones. 

As mentioned above, sodium-aluminum silicate paragonite and calcium-silicate hydrate, clinotobermorite formed only in the cracks of TA-modified sample cured in alkali carbonate. It is possible that clinotobermorite contained aluminum since its inclusion into tobermorite does not necessarily change the XRD pattern. Additionally gonnardite was possibly present in the cracks of TA-modified sample cured in alkali carbonate since its patterns largely overlap with those of thomsonite. 

In summary, the principal crystalline phases in TA-modified samples were poorer in calcium compared against the crystals in controls. The calcium-to-aluminum ratio of margarite preferentially crystalizing in TA-modified samples is lower than that of dmisteinbergite, predominant in control ones (1-to-4 ratio vs. 1-to-2 ratio, respectively), analcime, a major crystalline product in the material sealing the cracks in TA-samples does not contain any calcium, while peak intensities of calcium-containing cancrinite are higher for controls. This confirms earlier findings where minerals with lower calcium content formed in TA-modified samples after short curing times [44]. The lower calcium content of crystals suggests a possibility of higher calcium presence in the amorphous hydrates in TA-samples.

Crystalline phases identified by XRD cannot account for the high silicon content detected by µEDX. This suggests existence of amorphous silica in both control and TA-modified samples. 

### 3.4. DTG Study

The differential thermogravimetric analyses complimented the XRD studies for identification of both crystalline and amorphous phases that decompose with the weight loss at temperatures up to 1000 °C. The results are shown in Table 4. The weight losses below about 200 °C were attributed to poorly crystalline (sodium, calcium)-aluminosilicate hydrates and calcium-silicate hydrates [46,47,48] along with the water loss from zeolite-type phases such as gonnardite and possibly sodalite that showed minor peaks in XRD studies [49]. Analcime and thomsonite decompose for the most part in 200–400 °C temperature range, although increasing CaO content may shift analcime decomposition temperature up to 700 °C [50]. Garnet phases, such as grossular also have decomposition peaks in that temperature range [51,52,53]. Bohmite loses its hydroxyl groups between 400 °C and 550 °C. There is a possible contribution from thomsonite structure collapse above 500 °C [54]. Carbonates decompose at temperatures above 550 °C, including cancrinite decomposition at about 680 °C and 950 °C [55], iron calcium carbonate decomposition may take place in two steps (650–700 °C and 850–950 °C), sodium carbonate decomposes between 900 °C and 1200 °C. Mica group minerals decompose between about 820 and 920 °C [49]. 

For the water environment the major differences between control and TA-modified samples included: (1) higher weight losses in TA-modified samples up to 400 °C associated with amorphous and poorly crystalline hydrates (<200 °C) and zeolites and garnet phases (200–400 °C); and (2) lower losses in TA-samples from bohmite (400–550 °C). 

These results are in general agreement with the XRD-study that suggested higher zeolites content (analcime and thomsonite), grossular and smaller amount of aluminum oxide hydroxide (bohmite) in TA-modified samples. Additionally, it suggests more amorphous hydrates in composition of the TA-samples. 

It should be noted that the loss in 400–550 °C temperature range assigned to bohmite could be overestimated for TA-samples because of the possible contribution from zeolites. However, the fact that for both control and TA-samples the loss in that temperature range was larger in the core than in the crack areas suggests bohmite, which predominantly forms in cores, to be the main contributor to these losses. The decomposition temperature was lower for TA-modified samples in this range likely because of the lower crystallinity and possibly different nature of the decomposing phases (bohmite in the case of control, contribution of zeolites in the case of TA-samples).

The losses above 550 °C were assigned to carbonates and mica group mineral, margarite. The carbonate-related peaks at about 630 °C were larger for TA-modified samples in agreement with the XRD results. Large decomposition peaks at temperatures above about 750 °C for control samples may be associated with margarite. However, it is also possible that they are associated with the carbonation and further decomposition of Ca(Na)-Al-Si-H gel [56]. 

The weight losses of control and modified samples cured in carbonate were for the most part similar. The main difference was in noticeably higher weight losses above 800 °C for TA-samples. These are likely associated with the carbonation of cations originally bound to TA so removed from the pool of ions participating in formation of other minerals. Other contributors to this mass loss include cancrinite and margarite. The sample from TA crack area had two peaks at around 680 and 912 °C that are likely related to cancrinite or its precursor while the core sample had a wide peak above about 850 °C with probable contribution of margarite (not shown). 

Interpretation of specific peaks in complex blends of the samples is problematic. However, some general trends confirm XRD observations such as likely presence of zeolites, aluminum oxide hydroxides, margarite and cancrinite minerals in the healed samples. There are higher losses associated with amorphous phases and zeolites in TA-modified interface samples and lower losses from aluminum oxide hydroxides in the cores of these samples.

### 3.5. ATR-FTIR Studies

ATR-FTIR study was performed to support XRD results on the core and interface samples of specimens after 15 days of total healing and the third brake. The ATR-FTIR spectra of starting materials , CAC #80 and FAF (not shown), disclosed a very strong band at 770 cm^−1^ associated with the Al-O bond of “condensed” AlO_4_ tetrahedra network of mono-calcium aluminate (CA), the principal mineralogical phase of CAC [57,58]. The two major components of FAF were: the aluminosilicate anhydrous compounds showing the strong and weak bands at 1035 and 776 cm^−1^, respectively, belonging to the M-O (M: Si or Al) anti-symmetrical (V*_as M-O_*) and symmetric (V*_s M-O_*) stretching vibration in the Si-O-Si and Si-O-Al linkages [59,60]; the second component was quartz with weak 740 and 680 cm^−1^ bands attributed to Si-O symmetric stretching (V*_s Si-O_*) and Si-O bending (δ*_O-Si-O_*) vibrations, respectively [61,62]. 

**Figure 8:**
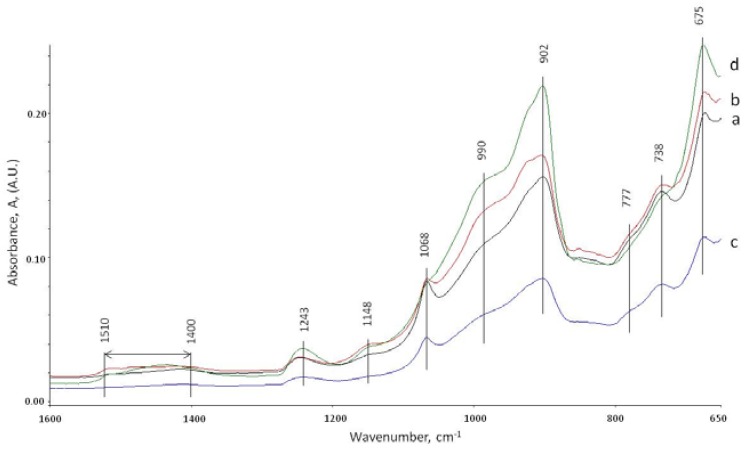
ATR-FTIR spectra of the: core (**a**); and crack (**b**) areas for control cements; and the: core (**c**); and crack (**d**) areas for TA-modified cements in alkali carbonate environment at 270 °C.

Figure 8 depicts the ATR-FTIR absorption spectra in the region of 1600 to 650 cm^−1^ for the core and inner-surface of cracks samples exposed to 270 °C alkali carbonate solution for 15 days. The spectral features closely resembled each other containing the same frequency nine representative frequency bands with the following contributors. The C-O (V*_as C-O_*) stretching vibration in carbonate CO_3_^2−^ emerged in the region of 1510 to 1400 cm^−1^ [63,64,65], the Si-O-Si linkage-associated V*_as Si-O_* vibration in silica gel at 1243 cm^−1^ [66,67,68], the Al-OH bending (δ*_as Al-OH_*) and (δ*_s Al-OH_*) vibrations, and AlO_4_ tetrahedral (V*_Al-O_*) stretching in bohmite (γ-AlOOH), were respectively, at 1148, 1068, and 777 cm^−1^, reflecting the presence of typical bohmite-related doublet peaks of O-H group at 3307 (V*_as O-H_*) and 3086 (V*_s O-H_*) cm^−1^ bands (not shown) [69,70,71,72], the M-O (M: Si or Al) (V*_as M-O_*) in amorphous and crystalline Na_2_O-Al_2_O_3_-SiO_2_-H_2_O (N-A-S-H), CaO-Al_2_O_3_-SiO_2_-H_2_O (C-A-S-H), and CaO,Na_2_O-Al_2_O_3_-SiO_2_-H_2_O (C,N-A-S-H) hydrates appeared at 990 and 902 cm^−1^ [73,74,48], and the Si-O stretching (V*_s Si-O_*) and Si-O bending (δ *_O-Si-O_*) modes in quartz at 738 and 675 cm^−1^, respectively with a possible quartz contribution to the band at 1068 cm^−1^ (V*_s Si-O_*) [62]. The N-A-S-H, C-A-S-H, and C,N-A-S-H phases were assembled by the interactions between Na^+^ and Ca^2+^ released by SMS activator and CAC, and the aluminosilicate anhydrous compounds in FAF [75]. The rest of the aluminum from CAC appeared in bohmite. The carbonates formed with the carbonate ion from the curing environment. Additional amorphous and crystalline silica showing prominent bands were derived from alkaline degradation of FAF and alkali carbonate environment as well as from the dissolution of SMS. 

Comparison of absorbance peak heights of the bands representative of carbonate, silica gel, bohmite, N-A-S-H, C-A-S-H, and C,N-A-S-H reaction products, and quartz for core and the inner crack surface areas of control and TA-modified samples in carbonate environment led to the identification of potential sealing and plugging reaction products. The cracks in TA-modified cement were plugged by carbonate-, silica-, N-A-S-H-, C-A-S-H-, and C,N-A-S-H-related products; in fact, the height of band peaks belonging to all these reaction products strikingly increased in the samples from crack surfaces, compared with the cores. In contrast, the bohmite-related peak height was reduced in the crack areas, so underscoring that the bohmite did not contribute significantly to the sealing of the cracks. This finding strongly supported the results from XRD study and is in agreement with the DTG analyses showing higher zeolite- and amorphous phases-related decomposition peaks for TA-modified samples. 

It is possible to rationalize that the principal reaction products have the highest peaks. Thus, the N-A-S-H-, C-A-S-H-, and C,N-A-S-H-related products are the major contributors to crack’s plugging. If this interpretation is valid, the secondary contributor was quartz, while the carbonates and silica gel role was minor. Unlike TA-modified cement, control cement was not as effective in plugging the cracks. In fact, the heights of the peaks were similar for the core and crack areas for all identified components. Accordingly, it appeared that TA served in the creation of the crack-plugging reaction products in alkali carbonate environment. Relating these findings to XRD data, the crystalline reaction products involved analcime and paragonite, in N-A-S-H system, cancrinite as carbonated-C,N-A-S-H, and dmisteinbergite in C-A-S system. In addition, tobermorite (or possibly aluminum-containing tobermorite) formed in the carbonate environment, contributing to the C-A-S-H peak.

**Figure 9:**
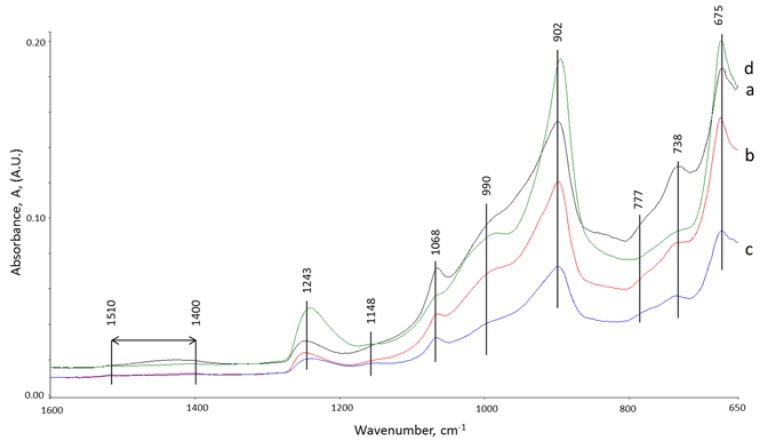
ATR-FTIR spectra of the: core (**a**); and crack (**b**) areas for control cements; and the: core (**c**); and crack (**d**) areas for TA-modified cements in water environment at 270 °C.

The overall appearance of the spectral features of the samples cured at 270 °C in water environment for total of 15 days was similar to that of the samples cured in carbonate (Figure 9). Similar to the carbonate environment for TA-modified samples, the heights of the peaks of N-A-S-H-, C-A-S-H-, and C,N-A-S-H-related products, silica gel and quartz were higher in the crack areas than in the core samples.

Thus, there was no doubt that although the water environment differed from alkali carbonate, TA favorably promoted the formation of these reaction products in crack areas. However, despite this fact, the recovery rate of strength in water was lower than that in the carbonate, suggesting that the formation of carbonate compounds may be required to achieve a better strength recovery. 

The crack-plugging effects by the reaction products formed in TA-retarded cement exposed to alkali carbonation environment appeared to play a pivotal role in enhancing the recovery rate of compressive strength for the repeatedly crushed cements. 

### 3.6. Microstructural Characterization

**Figure 10:**
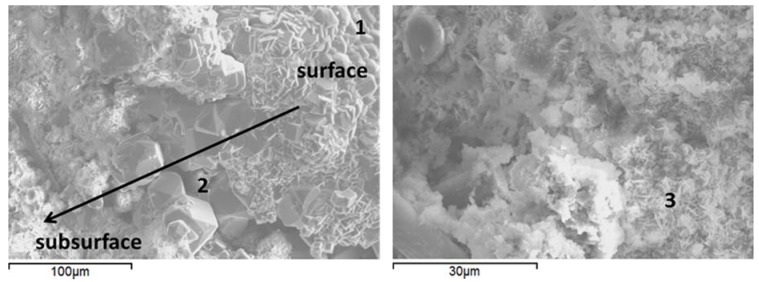
Scanning electron micrographs and point EDX data of typical cement microstructures for control specimens cured in: water (**left**); or alkaline carbonate (**right**) at 270 °C.

**Figure 11:**
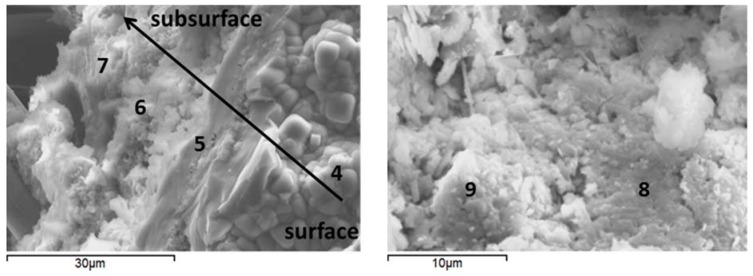
Scanning electron micrographs and point EDX data of typical cement microstructures for TA-modified specimens cured in alkali carbonate at 270 °C.

To observe the morphological features and support the XRD findings, the SEM-EDX studies were conducted on freshly fractured samples. The samples of crack surfaces and the undelaying areas were examined after the total of 15 days of healing in water or alkaline carbonate at 270 °C (Figure 10 and Figure 11, and Table 5 for point compositions). 

Morphological features of control and TA-modified samples were generally similar with the major difference being a lower crystallinity of TA-modified samples compared against control ones. The samples’ surfaces were composed of agglomerates, likely precipitated from the interstitial solution [76] (Figure 10 and 11 left, points 1 and 4). The silicon-surface content was clearly higher compared against that of the core for all the samples in agreement with EDX and FTIR measurements. With increasing distance from the surface, analcime cubic crystals were visible under the precipitated surface agglomerates in control samples (Figure 10 left, point 2). The cores of these samples included areas with elevated calcium typical for feldspar minerals (Figure 10 right, point 3) confirming the XRD data.

For TA-modified samples, the subsurface hydrates were more amorphous with growing aluminum content further from the surface (Figure 11 left, points 5–7).

The amorphous cores of TA-modified samples showed calcium included into the gel composition (Figure 11 right, point 8), which agrees with the lower calcium content of the crystalline hydrates in these specimens suggested by the XRD study. Some units with zeolite-type composition forming on fly ash particles were identified in the cores of these samples (Figure 11 right; point 9).

## 4. Discussion

A previous work demonstrated that TA alters cement phase compositions, improves compressive strength development and increases the number of pores below 500 microns after three days of curing at temperatures up to 300 °C [44]. The present study confirms that some differences in TA-modified cements remain after longer curing times of 15 days at 270 °C. Specifically, the compressive strength of the retarded samples remains higher compared against control, the predominant crystalline phases have lower calcium content (analcime and margarite vs. dmisteinbergite) and the general crystallinity of modified samples remains lower. 

The retarder also changes self-healing properties of the cement that undergoes compressive damage with the crack formation. It affects both crack-plugging ability and strength regaining properties, which are superior for the retarded slurries. 

High temperature hydration of TSRC results in calcium-aluminum silicates, zeolite-family crystalline products and (C,N)-A-S-H amorphous phase [43,73]. The crystalline calcium-aluminum-silicate hydrates include hydrogrossular phases at earlier hydration times (katoite, grossular, and hibschite) and feldspar (dmisteinbergite and anorthite) or feldspathoid (cancrinite in carbonate-rich environment) type minerals after longer curing at temperature above about 250 °C. Tartaric acid that acts as a set retarder for TSRC slurries changes the early-time phase composition favoring fewer calcium- and aluminum- rich phases such as feldspar minerals, bohmite and more high-temperature stable zeolites in the set cement [44]. Calcium and aluminum interactions with tartaric acid in the first minutes of hydration decrease their availability for crystals formation [77]. These cations eventually are included into amorphous (C,N)-A-S-H gel, which even at 270 °C is among the major phases of hydrated TSRC [43]. Kinetics of gel crystallization into zeolites are generally very slow; in time zeolites such as thomsonite and especially high-temperature stable analcime crystalize from the gel phase. 

If this hypothesis is correct, the first-minute interactions in slurries are critically important for the phase composition of the set cement. Thus, cation-binding molecules such as tartaric acid effectively remove certain cations from the early developing crystals and transport them into the amorphous phase that forms later. In addition to tartaric acid any other cation-binding molecules from the environment may affect the solid phase composition of the hydrating blend. Carboxylic acid anions in CO_2_-rich environments may effectively bind calcium ions and, similar to tartaric acid, interfere with crystallization of calcium-containing feldspar minerals promoting formation of feldspathoids in their place [44]. Silicon from fly ash and sodium meta-silicate activator precipitates as silicon-rich gel with calcium, sodium and aluminum ions reserved through earlier interactions with TA. Carbonate environment especially favors silica precipitation lowering its solubility or assisting in its release from calcium-aluminum silicate phases through calcium dissolution by formation of soluble calcium bicarbonates. Inclusion of calcium and aluminum cations changes the structure and stability of this silicon-rich amorphous phase. At longer curing periods zeolites crystallize from the silica-aluminum-rich amorphous gel. 

There is a general agreement between the experimental results. XRD, ATR-FTIR and DTG show larger zeolites and limited bohmite presence in cements modified with tartaric acid. ATR-FTIR data also indicate higher abundance of (C,N)-A-S-H type products in these samples. XRD data suggest restricted crystallization of calcium-rich phases in modified samples. SEM morphological measurements confirm lower crystallinity with fewer calcium-rich feldspar type crystals in the presence of TA. Finally, EDX results clearly point out silicon enrichment at the surface of both control and TA-modified samples especially in the crack areas. Combining all these evidences the following strength recovery and sealing mechanism may be proposed. The binding phase providing matrix strength and fractures sealing is mostly amorphous of general (C,N)-A-S-H composition. Aluminum presence in the silicate gel increases the cross-linking in-between silicate chains [76]. In TA-modified samples, especially in carbonate environments, in addition to aluminum calcium joins the amorphous products. Both calcium and aluminum strengthen the silicate gel structure. Aluminum incorporation in the C-S-H gel was reported to entail formation of 3D aluminum-silicate skeletons with partial healing effect in the C-A-S-H nanostructure potentially increasing durability and strength of the hydration product [78,79]. Aluminum was also reported to accelerate formation and stabilize tobermorite at temperatures that are normally above its stability domain [80]. The crystallization of tobermorite in carbonate environment in TA-modified samples is in agreement with this hypothesis. In fact, grossular presence in this sample suggested by XRD data also confirms this argument since at high aluminum content grossular crystalizes from C-A-S-H gels. At longer curing times zeolites crystallize from the amorphous phase.

In summary, TA favors formation of amorphous hydrates rich in silicon with calcium and aluminum inclusions strengthening the gel and crystallization of zeolites. In the case of a carbonate environment where calcium may additionally solubilize through formation of calcium bicarbonate and precipitate later with the silicate, eventual crystallization of tobermorite in the fractures takes place, contributing not only to the sealing but also helping to bind the broken cement pieces to the matrix. 

## 5. Conclusions

The main results of this study on the role of tartaric acid in self-healing behavior of an alkali-activated blend of calcium aluminate cement and fly ash F (TSRC) in water or alkali carbonate at 270 °C are as follows.

1. Tartaric acid improves the initial strength of set cement and strength recovery and fractures sealing of TSRC both in water and alkali carbonate. The initial strength of TA-modified samples was 32 and 23% higher after 24 hours of curing in water and carbonate, respectively, than for control samples. After two crush tests and two recovery periods of 10 days followed by an additional five days at 270 °C, the strength recovery rates were the highest for the TA-modified samples healed in alkali carbonate and the lowest for the control samples healed in the same environment.

2. Crystalline phases with lower calcium content preferentially formed in TA-modified cement, including zeolite-type minerals, crystallization of bohmite was restricted by the tartaric acid. The subsurface areas and the matrix were more amorphous with fewer crystals for the modified samples. 

3. (C,N)-A-S-H phase predominant in TA-modified samples mostly contributed to the strength recovery and fractures sealing. The superior strength recovery and crack sealing in carbonate environment suggests importance of carbonate compounds in the healing process. It appears that TA served in the creation of the crack-plugging reaction products in this environment. Cations removal through binding to the tartaric acid and carbonates at early hydration times seems to help strength recoveries and cracks sealing possibly through formation of cross-linked sodium-calcium-aluminum-silicate gel, crystallization of carbonated compounds and tobermorite in alkali carbonate. Silica also contributed to the crack sealing. 

## Figures and Tables

**Table 1:** The elemental composition of Thermal Shock Resistant Cement components in mass fractions.

Component	Calcium Aluminate Cement	Flay Ash class F	Sodium Metasilicate
Al_2_O_3_	73.8	34.8	-
CaO	26.1	2.7	-
SiO_2_	-	50.4	46.6
Fe_2_O_3_	0.1	7.1	-
Na_2_O	-	0.3	50.5
K_2_O	-	3.1	-
TiO_2_	-	1.6	-

**Table 2:** Major oxides composition in the cracks and cores of the healed Thermal Shock Resistant Cement (TSRC) samples with or without tartaric acid (TA).

Sample	SiO_2_	Al_2_O_3_	CaO	CaO/Al_2_O_3_	CaO/SiO_2_	Al_2_O_3_/SiO_2_
***Samples from Healed Cracks***						
**Control Water**	78	11	3.9	0.35	0.05	0.14
**Control Carbonate**	79	11	5.6	0.51	0.07	0.14
**TA Water**	69	19	3.2	0.17	0.05	0.27
**TA Carbonate**	81	13	3.2	0.25	0.04	0.16
***Core Samples***						
**Control Water**	22	51	19	0.37	0.86	2.3
**Control Carbonate**	22	52	20	0.38	0.91	2.4
**TA Water**	23	51	19	0.37	0.83	2.2
**TA Carbonate**	23	52	18	0.35	0.78	2.3

**Table 3:** Main crystalline phases identified by XRD with corresponding The International Center for Diffraction Data (ICDD) numbers.

Control	TA-Modified
*Core—Water*	
04-011-5220 Dmisteinbergite CaAl_2_Si_2_O_8_; 04-013-3004 Margarite-2M1 CaAl_4_Si_2_O_10_(OH)_2_; 01-078-0296 Thomsonite-Ca NaCa_2_Al_5_Si_5_O_20_(H_2_O)_6_; 04-012-1749 Analcime Na_7.6_Al_7.6_Si_16.4_O_48_(H_2_O)_8_; 04-014-1755 Tohdite Al_5_O_7.5_(H_2_O)_0.5_; 00-005-0190 Bohmite Al_2_(OOH)_2_; 00-011-0401 Hydroxysodalite Na_4_Al_3_Si_3_O_12_OH	04-013-3004 Margarite-2M1 CaAl_4_Si_2_O_10_(OH)_2_; 01-078-0296 Thomsonite NaCa_2_Al_5_Si_5_O_20_(H_2_O)_6_; 04-011-5220 Dmisteinbergite CaAl_2_Si_2_O_8_; 04-013-2153 Grossular Ca_3_Al_2_(SiO_4_)_2_(OH)_4_; 01-072-0445 Analcime Na(AlSi_2_O_6_)(H_2_O)
*Crack—Water*	
04-011-5220 Dmisteinbergite CaAl_2_Si_2_O_8_; 01-076-6569 Analcime Na_0.9_((Al_0.9_Si_2_)O_6_(H_2_O); 04-013-3004 Margarite-2M1 CaAl_4_Si_2_O_10_(OH)_2_; 04-014-1755 Tohdite Al_5_O_7.5_(H_2_O)_0.5_; 00-005-0190 Bohmite Al_2_(OOH)_2_	00-018-0276 Margarite-2M1 CaAl_4_Si_2_O_10_(OH)_2_; 01-088-2093 Thomsonite-Ca Na_1.26_Ca_1.74_(Si_5.26_Al_4.74_O_20_)(H_2_O)_6_; 04-011-6755 Analcime Na_8_Al_8_Si_16_O_48_(H_2_O)_8_; 04-014-1755 Tohdite Al_5_O_7.5_(H_2_O)_0.5_; 04-011-5220 Dmisteinbergite CaAl_2_Si_2_O_8_; 04-013-2153 Grossular Ca_3_Al_2_(SiO_4_)_2_(OH)_4_
*Core—Alkali Carbonate*	
00-018-0276 Margarite-2M1 CaAl_2_(Si_2_Al_2_)O_10_(OH)_2_; 04-011-6755 Analcime Na_8_Al_8_Si_16_O_48_(H_2_O)_8_; 01-080-6030 Cancrinite Na_7.4_Ca_0.54_(Si_6.42_Al_5.58_O_24_)(CO_3_)_1.47_(H_2_O)_2_; 04-011-5220 Dmisteinbergite CaSi_2_Al_2_O_8_; 04-013-2153 Grossular Ca_3_Al_2_(SiO_4_)_2_(OH)_4_; 00-005-0190 Bohmite Al_2_(OOH)_2_	00-018-0276 Margarite-2M1 CaAl_2_(Si_2_Al_2_)O_10_(OH)_2_; 04-011-7963 Analcime-R Na_8_Al_8_Si_16_O_48_(H_2_O)_8_; 01-078-0296 Thomsonite-Ca NaCa_2_Al_5_Si_5_O_20_(H_2_O)_6_; 01-075-8619 Cancrinite Na_6.02_Ca_1.52_(Si_6_Al_6_O_24_)(CO_3_)_1.52_(H_2_O)_1.56_; 04-011-5220 Dmisteinbergite CaSi_2_Al_2_O_8_; 04-013-2153 Grossular Ca_3_Al_2_(SiO_4_)_2_(OH)_4_; 04-014-1755 Tohdite Al_5_O_7.5_(H_2_O)_0.5_
*Crack—Alkali Carbonate*	
04-013-1895 Cancrinite Na_6_Ca_1.5_Al_6_(SiO_4_)_6_(CO_3_)_1.5_(H_2_O)_1.75_ 04-011-7963 Analcime-R Na_8_Al_8_Si_16_O_48_(H_2_O)_8_; 04-015-7167 Coesite SiO_2_; 00-018-0276 Margarite-2M1 CaAl_4_Si_2_O_10_(OH)_2_; 04-011-5220 Dmisteinbergite CaSi_2_Al_2_O_8_; 00-005-0190 Bohmite Al_2_(OOH)_2_	01-074-2596 Clinotobermorite Ca_5_(Si_6_O_16_)(OH)_2_; 04-011-7963 Analcime-R Na_8_Al_8_Si_16_O_48_(H_2_O)_8_; 01-075-8620 Cancrinite Na_6.02_Ca_1.52_(Al_6_Si_6_O_24_)(CO_3_)_1.52_; 04-011-5220 Dmisteinbergite CaSi_2_Al_2_O_8_; 01-082-2720 Paragonite-2M1 Na_4_Al_12_Si_12_O_40_(OH)_8_

**Table 4:** Mass losses in different temperature ranges for the 15-day cured interface (IF) and core samples.

Specimen	Decomposition Temperature Range, °C									
	<200 Ca(Na)-Al-Si-H Clinotobermorite (Zeolites)		2004400 Zeolites Grossular		400–550 Bohmite (Zeolites)		>550 Carbonates Mica group		Total	
	crack	core	crack	core	crack	core	crack	core	crack	core
**Carbonate-Control**	1.4	1.5	2.1	2.0	1.9	2.4	3.2	2.2	8.6	8.0
**Carbonate-TA**	1.1	1.5	2.3	1.9	1.2	2.3	9.4	7.1	14.1	12.8
**Water-Control**	0.94	1.3	1.6	1.5	1.3	2.2	7.5	7.1	11.4	12.0
**Water-TA**	1.5	1.7	2.1	1.9	1.6	2.1	2.2	4.2	7.3	9.9

**Table 5:** Point compositions of typical cement microstructures shown in Figure 10.

Sample	Point	Al	Ca	K	Na	Si	Possible phase
**Water-Control**	1	5.89	0.33	1.85	1.28	26.65	Si-rich gel
	2	9.61	0.53	0.16	4.68	22.55	Analcime
**Carbonate-Control**	3	24.86	4.11	0.23	1.3	9.12	Al-rich gel, dmisteinbergite precursor
**Carbonate-TA**	4	6.15	0.14	1.17	3.71	25.68	Si-rich gel
	5	10.93	1.62	0.1	6.0	20.09	Si-rich gel
	6	13.38	3.6	0.25	4.14	17.58	Si-Al-rich gel
	7	23.23	3.13	0.78	1.19	9.51	Ca(Na)-Al-Si gel
	8	20.75	5.97	0.21	1.58	11.16	Ca(Na)-Al-Si gel
	9 ^1^	10.11	1.53	0.24	14.93	7.20	Carbonated zeolite or cancrinite precursor

^1^ – 9% carbon in composition.

## Abbreviations

The following abbreviations are used in this manuscript:

TA: Tartaric Acid
CAC: Calcium Aluminate Cement
FAF: Fly Ash type F
SMS: Sodium Meta-Silicate
TSRC: Thermal Shock Resistant Cement
